# Editorial: Mitophagy in Health and Disease

**DOI:** 10.3389/fcell.2021.647036

**Published:** 2021-02-01

**Authors:** Konstantinos Palikaras, Evandro Fei Fang, Nektarios Tavernarakis

**Affiliations:** ^1^Institute of Molecular Biology and Biotechnology, Foundation for Research and Technology-Hellas, Heraklion, Greece; ^2^Department of Basic Sciences, School of Medicine, University of Crete, Heraklion, Greece; ^3^Department of Clinical Molecular Biology, University of Oslo and Akershus University Hospital, Lørenskog, Norway; ^4^The Norwegian Centre on Healthy Ageing (NO-Age), Oslo, Norway

**Keywords:** aging, autophagy, cell death, energy metabolism, homeostasis, mitochondria, mitophagy, neurodegeneration

Mitophagy is the major degradation pathway, by which cells regulate mitochondrial number and integrity, in response to metabolic and physiological state. Research in mitophagy has grown exponentially over the last decade, revealing the intricate signaling pathways regulating mitophagy and the complexities of the molecular machinery involved in carrying out mitochondrial elimination. A diverse repertoire of mitophagy-related proteins has been discovered, highlighting an elaborate regulatory network of mitochondrial homeostasis that responds differentially to developmental, hormonal, and/or environmental signals. Moreover, the multi-layered crosstalk between mitophagy signaling pathways sustains energy metabolism, which is critical for tissues and organs homeostasis. Indeed, defective mitophagy causes accrual of dysfunctional mitochondria leading to bioenergetic stress, elevated ROS levels and pronounced inflammation that is accompanied by cellular and tissue degeneration. Thus, mitophagy is a pivotal contributor to cellular physiology, and tissue integrity, in addition to organismal development, healthspan, and survival.

The Research Topic on “Mitophagy in Health and Disease” in *Frontiers in Cell and Developmental Biology* includes a series of 11 articles that discuss recent advances in the field of mitophagy research and highlight challenges and outstanding questions, that need to be addressed before mitophagy modulation can be considered for the development of effective therapeutic interventions.

Several molecular mechanisms have been identified that mediate mitochondrial removal in a cell type- and tissue-specific manner. In their review, Ravanelli et al. discuss the critical role of the ubiquitin/proteasome system (UPS) in mitochondrial quality control. Alterations in the ubiquitination status of mitochondrial proteins contribute to the remodeling of the mitochondrial proteome in response to stress conditions. The authors discuss the tight crosstalk between the UPS and mitochondria that contributes to prevent proteostasis collapse and promote energy metabolism.

The article by Wang et al. surveys the regulation of mitochondrial removal by the phosphatase and tensin homolog (PTEN) isoforms. The authors introduce the molecular function of PTEN-short and PTEN-long proteins and their association with the mitophagic machinery. In addition, the authors highlight post-translational modifications as a central node of mitophagy, suggesting that their modulation can be used for the development of novel intervention approaches toward tackling mitochondrial-related disorders.

In their review, Ravanidis and Doxakis address the role of RNA-binding proteins (RBPs) in mitochondrial homeostasis. The authors describe the vital role of RBPs in the maintenance of mitochondrial metabolism through the regulation of mRNA splicing, stability, targeting to mitochondria and translation. Importantly, substantial evidence indicates that impaired expression, or mutations in RBPs, contribute to mitochondrial dysfunction that has been implicated in the development and progression of several age-associated neurodegenerative disorders.

Although several components of the mitophagic machinery have been uncovered, the origin of mitoautophagosomal membranes remains elusive. In their review, Zachari and Ktistakis survey the molecular mechanisms that govern mitochondrial degradation, with a particular focus on the early signaling events of mitophagy initiation and mitoautophagosome formation.

A growing body of evidence suggests an intricate communication between mitophagy and cell death pathways. In their review, Ma et al. focus on how excessive mitochondrial damage can trigger innate immune responses and apoptotic cell death via BCL2 protein family members. The authors explore the molecular mechanisms that uphold mitochondrial homeostasis, including mitochondrial dynamics, mitochondrial biogenesis, and mitophagy among others, as well as, how these cellular events interfere with cell fate. Moreover, a relevant review by Joaquim and Escobar-Henriques discusses the pro-survival and pro-apoptotic role of mitophagy. The authors describe the involvement of mitofusins in mitophagy, through the modulation of mitochondrial morphology and endoplasmic reticulum (ER)-mitochondria contact sites. Finally, they summarize emerging findings, suggesting that impaired mitochondrial dynamics and mitophagy contribute to the pathogenesis of non-alcoholic liver disease.

Several studies have revealed a progressive, age-related decline of mitophagic flux in multiple tissues, including as heart, kidney, liver, and brain. In their article, Luo et al. discuss the contribution of mitophagy deregulation during aging to the homeostasis and viability of post-mitotic neurons and cardiomyocytes. Furthermore, the authors discuss recent studies that link impaired mitophagy to the development of neurodegenerative and cardiovascular pathologies.

The article by Xie C. et al. survey the role of defective mitophagy in the pathogenesis of Alzheimer disease (AD). Increasing evidence indicates that accumulation of damaged mitochondria, due to mitophagy impairment contributes to Aβ/Tau proteinopathies and stimulates persistent inflammation, causing neuronal loss, and cognitive decline. In addition, the authors discuss the potential use of mitophagy inducers, such as NAD^+^ precursor molecules and urolithin A, toward ameliorating aging, and AD pathological features.

The review by Xie Y. et al. surveys the molecular pathways that govern mitochondrial elimination, focusing on the essential role of mitophagy receptors. Furthermore, the authors discuss the involvement of mitophagy receptors in tumorigenesis, and highlight the therapeutic potential of mitophagy modulation in cancer therapies.

The last two articles discuss emerging findings that highlight the anti-aging properties of mitophagy. In their review, Chen et al. delineate the molecular pathways and mechanisms of mitophagy in several model organisms, and discuss the significant contribution of mitophagy defects in age-related pathologies. In their article, Bakula and Scheibye-Knudsen introduce the term “mitophaging” pointing to the fundamental role of mitochondrial integrity in the maintenance of cellular fitness and organismal health. Both articles discuss interventions that target different steps in the process of mitophagy, by utilizing small molecular compounds as a means toward the development of novel, effective treatments against currently incurable pathologies.

In closing this Editorial piece, we would like to thank all the authors and referees, for their valuable contributions, toward compiling this up-to-date and timely Research Topic on mitophagy in health and disease. Moreover, we hope that the collection of articles included in the topic will provide a useful point of reference and a stimulus for further research aiming to ultimately decipher the complex contributions of mitophagy to cellular and organismal homeostasis.

## Author Contributions

KP and NT wrote the manuscript. KP, EF, and NT read and edited the manuscript. All authors contributed to the article and approved the submitted version.

## Conflict of Interest

EF has CRADA arrangement with ChromaDex, and is consultant to Aladdin Healthcare Technologies, Vancouver Dementia Prevention Centre, and Intellectual Labs. The remaining authors declare that the research was conducted in the absence of any commercial or financial relationships that could be construed as a potential conflict of interest.

